# In touch: Cardiac and respiratory patterns synchronize during ensemble singing with physical contact

**DOI:** 10.3389/fnhum.2022.928563

**Published:** 2022-08-05

**Authors:** Elke B. Lange, Diana Omigie, Carlos Trenado, Viktor Müller, Melanie Wald-Fuhrmann, Julia Merrill

**Affiliations:** ^1^Music Department, Max Planck Institute for Empirical Aesthetics, Frankfurt am Main, Germany; ^2^Department of Psychology, Goldsmiths, University of London, London, United Kingdom; ^3^Center for Lifespan Psychology, Max Planck Institute for Human Development, Berlin, Germany; ^4^Max Planck NYU Center for Music, Language, and Emotions, Frankfurt am Main, Germany; ^5^Institute of Music, University of Kassel, Kassel, Germany

**Keywords:** joint action, HRV, respiration, singing ensemble, polyphonic music, supersubject, hyperscanning

## Abstract

Musical ensemble performances provide an ideal environment to gain knowledge about complex human interactions. Network structures of synchronization can reflect specific roles of individual performers on the one hand and a higher level of organization of all performers as a superordinate system on the other. This study builds on research on joint singing, using hyperscanning of respiration and heart rate variability (HRV) from eight professional singers. Singers performed polyphonic music, distributing their breathing within the same voice and singing without and with physical contact: that is touching each other's shoulder or waist. The idea of singing with touch was motivated by historical depictions of ensemble performances that showed singers touching each other. It raises the question of the potential benefit of touch for group performances. From a psycho-physiological point of view, physical contact should increase the synchronization of singing coordination. The results confirm previous findings on synchronization of respiration and HRV during choir singing and extend those findings to a non-homophonic musical repertoire while also revealing an increase in synchronization in respiration during physical contact. These effects were significant across different frequency ranges. The effect of physical contact was stronger when all singers were singing in comparison to the partial ensemble. Importantly, the synchronization could not be fully explained by the singing action (i.e., singing the same voice, or singing vs. listening) or by the standing position or touch. This finding suggests a higher level of organization of all singers, forming a superordinate system.

## Introduction

Joint human interaction requires highly synchronized behavior to achieve individual or group goals (Valdesolo et al., 2010; Konvalinka et al., 2011). Interactions have often been investigated in dyadic constellations (Konvalinka and Roepstorff, 2012), for instance, to better understand unidirectional effects (Goldstein et al., 2017), sender-receiver relations (Montague et al., 2002), leader-follower relations (Konvalinka et al., 2011; Sänger et al., 2012), and also to investigate ongoing, real-time, mutual coordination (Tognoli et al., 2007; Lindenberger et al., 2009). Importantly, interacting entities are not only separate units but are coupled (Konvalinka and Roepstorff, 2012). Systematic research on the coordination of larger groups is still in its beginnings and has often occurred in a musical context (Babiloni et al., 2011, 2012; Müller and Lindenberger, 2011, 2019; Müller et al., 2013, 2018a,b, 2019; Vickhoff et al., 2013; Glowinski et al., 2015; Osaka et al., 2015; Hemakom et al., 2016, 2017; Kaneshiro et al., 2016). To gain knowledge about complex human interaction, investigating musical performance might be ideal because of the inherent variety of individual and group goals. In ensemble music, musicians have to adapt their individual voices within the musical context and in relation to the interpretation of the other voices. This requires a constant adaptation of own and joint goals in terms of tempo, intensity, and timbre to arrive at a joint and coherent musical interpretation of a piece.

Coordination between musicians performing in ensembles has been shown on different levels. For example, on the behavioral level, head movements cue musical structure and different performance practices (Glowinski et al., 2015; Bishop et al., 2019). Interestingly, coordination has also been demonstrated on the level of physiological processes that are less obvious, for example, respiratory and cardiac responses (Müller and Lindenberger, 2011; Vickhoff et al., 2013; Hemakom et al., 2016, 2017; Müller et al., 2018a, 2019), and brain responses (Lindenberger et al., 2009; Babiloni et al., 2011, 2012; Sänger et al., 2012; Müller et al., 2013, 2018b, 2019; Osaka et al., 2015), suggesting that coordination extends to implicit processes. Neural and physiological oscillations have shown inter-person and intra-person couplings and have revealed an underlying complex network structure within and between brains (Müller et al., 2018b, 2021). On the one hand, it is conceivable that these network structures reflect specific roles of individual musical performers, and on the other hand, a higher level of organization of all performers as a superordinate system (Noble, 2012) or superorganism.

Our study builds on previous research on joint singing, using hyperscanning (e.g., simultaneous recording of several psychophysiological measures from several participants; see Müller et al., 2021) of respiration and HRV from an ensemble of eight singers. Studies have demonstrated increased phase synchronization of respiration and HRV during singing in comparison to a resting condition (Müller and Lindenberger, 2011) or breaks during a concert (Hemakom et al., 2017). Phase synchronization was higher in a choir during singing in unison, in comparison to singing a canon in parts (Müller and Lindenberger, 2011). However, singing in canon resulted in the coupling of singers singing the same voice,^1^ revealing a modular structure based on the musical score (Müller and Lindenberger, 2011). Coupling between singers has been shown to be higher than between members of the audience (Hemakom et al., 2016), suggesting that the act of singing results in more synchronized physiological processes than simply perceiving music.

However, synchronized respiration in monophonic and homophonic music may not seem very surprising, given that singing is uniquely related to a characteristic use of respiration. The exhaled air vibrates the vocal cords, and the controlled adjustments of the resonance apparatus and articulators result in different timbres (Kang et al., 2018). Breathing has to be coordinated with the musical progression and musical phrase endings or breaks, offering suitable time points for breathing. Further, as respiration and heart rate are coupled physiological signals (e.g., suppression of heartbeat during exhalation), synchronized breathing can also result in synchronization of HRV. Indeed, strong relations between synchronized breathing during singing and coupled HRV have been demonstrated, when comparing three conditions (Vickhoff et al., 2013): singers hummed a tone without breathing instructions and the tone did not include musical structure, or singers sang a hymn at a specific tempo related to 0.2 Hz, or, finally, they sang a mantra with breathing instructed at 0.1 Hz in relation to the musical structure (breaks). During humming, singers showed inter-individual differences for specific periodicities within respiration and HRV. While singing the hymn, respiration and HRV synchronized at 0.2 Hz (as well as at 0.05 and 0.1 Hz). Singing the mantra resulted in the strongest synchronization across conditions with a peak at 0.1 Hz. In other words, singing the same musical voice coordinates breathing which, in turn, results in systematic synchronization of respiration frequencies. However, turning back to the results of Müller and Lindenberger (2011), singing songs in parts and canons resulted in synchronized respiration and HRV on several oscillation frequencies, suggesting that synchronization is not limited to frequencies inherent in the musical structure.

We follow up on previous studies on singing with the goal of replicating and extending results on synchronization of respiration and HRV. A professional ensemble performed polyphonic a-cappella music from the Renaissance, which can be considered one of the most complex and intricate forms of European multi-part music. Each voice typically has an identity of its own: specifically, the beginning and end of phrases often overlap between voices and do not happen simultaneously. The voices are woven into each other, blending into a continuous stream of a musical sound. To achieve an uninterrupted flow of musical sound, professional choirs make use of distributed breathing, avoiding breathing at the same time (see Supplementary Figure 1 depicting no inter-subject synchronization of the audible breathing onsets in our data sets). Our first research question was, can we replicate the synchronization of respiration and HRV even when the sung music is polyphonic, i.e., when the phrases of the musical voices are mostly independent of each other?

**Figure 1:**
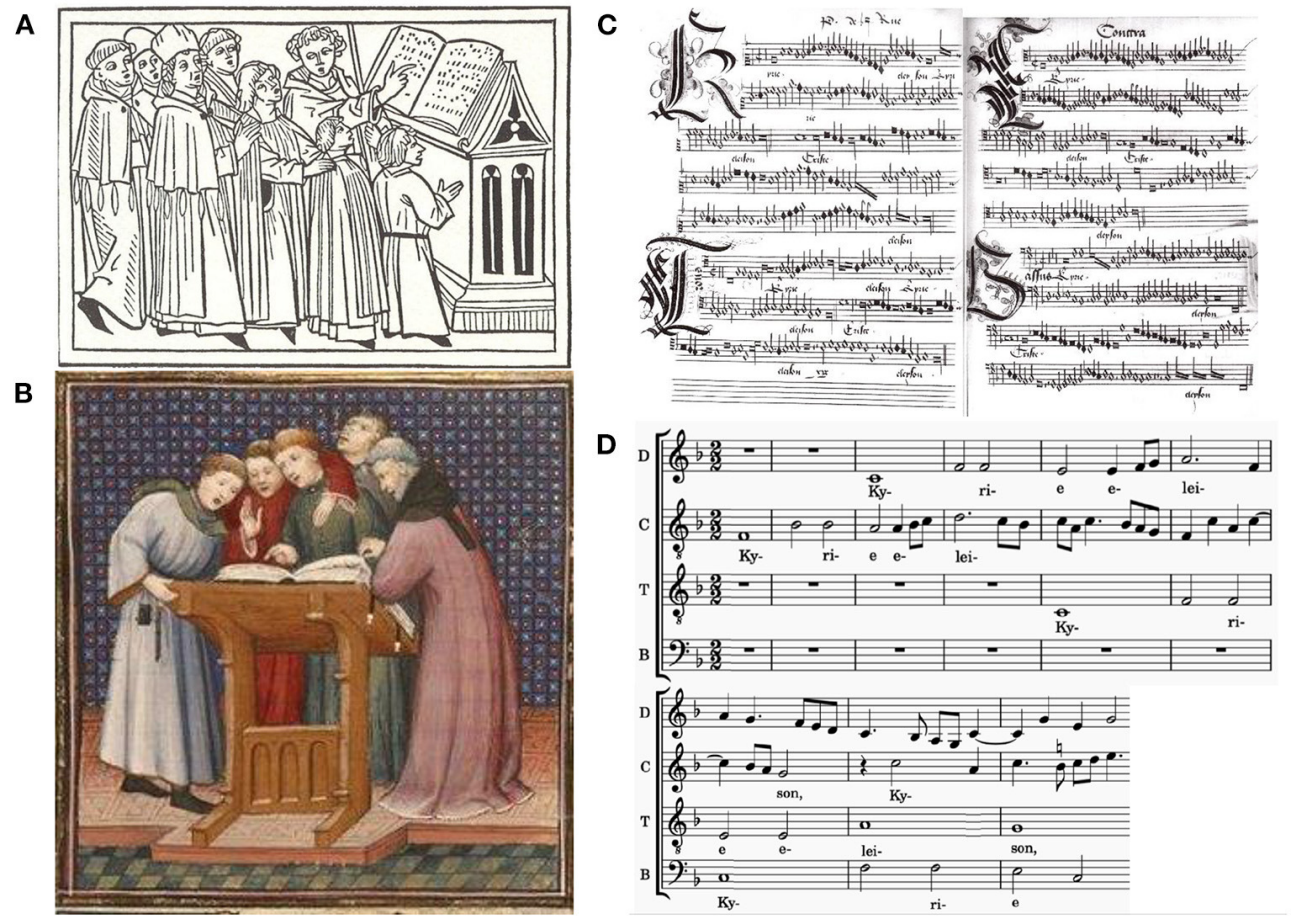
**(A)** Initial to the entry “Cantus,” Rodericus Zamorensis, Speculum vitae humanae, 1468; **(B)** Miniature at the beginning of Psalm 150, Grande bible historiale complétée (1395–1401), Maître du livre d'heures de Johannette Ravanelle, Paris, Bibliothèque nationale, Ms. fr. 159, f. 277v. (source: gallica.bnf.fr / BnF); **(C)** Mensural (Historic musical) notation of Pierre De la Rue: *Missa Almana, Kyrie* (before 1518) with the four voices separately in blocks, upper left, upper right, lower left, lower right (source: Brüssel, Koninklijke Bibliotheek van Belgie, ms. 9126, 58v und 59r.); **(D)** Modern notation of the same composition with the four voices in horizontal lines and the vertical indicating time.

We further extended the study to include a feature that is often shown in pictorial representations of singing and other music ensembles from the fourteenth to seventeenth centuries. Singing ensembles are displayed standing close and even in an embrace and with physical contact (Tammen, 2013). An investigation of historical sources resulted in a large corpus of these depictions (Wald-Fuhrmann et al., 2014; Max Planck Institute for Empirical Aesthetics, 2017). Embraces between members of a chapel seem to be unusual. They were required to cross the upper arms in front of their breast, above or below the mantle. Also, almost no other historical sources exist that corroborate that singing with physical contact was actually a common feature of the performance practices of that time. However, in the context of Christian sacred music, some reasons for physical contact are plausible: For example, singing from only one music book that was placed on a large note stand made standing close and eventually physical contact unavoidable (Figures 1A,B). In addition, physical contact might benefit keeping time: The mensural notation system in which this repertoire was written down did not provide any visual cues for temporal coordination across voices in polyphonic compositions. The four or more voices were notated in individual blocks, e.g., the upper left quadrant showed notation for the Superius, upper right Altus, lower left Tenor, and lower right Bassus (Figure 1C). In modern notation, voices are ordered in horizontal lines, and bar lines are used as a visual cue for temporal alignment (Figure 1D). In most historical depictions, physical contact is applied either by putting the hand on the shoulder or head of the singer in front, or the arm around the shoulder of a neighboring singer, i.e., contact is established between singers of the same as well as of different voices. Sometimes, the position of the pointing finger invites the interpretation that part of the contact was to periodically tap on the shoulder to provide an external pacemaker in the absence of a conductor, an aspect of historical performance practice for which at least scarce literary sources exist (Frobenius, 1972). Given the complexity of polyphonic music and its traditional notation, however, additional means for temporally coordinating the singers' actions might have been used. If an effect of touch on singers and singing were to be shown, this could indicate that physical contact might have been one such additional means. It should be noted, however, that instead of a literal understanding of the depictions, several other interpretations are conceivable, too. The hand on the shoulder might symbolize the unifying forces of musical performance, the group identity of the clergy, the harmonious character of their performance, or an act of consecration (Tammen, 2013).

From a psycho-physiological point of view, physical contact can indeed be expected to increase the synchronization of singing coordination on several levels, for example by increasing the synchronization of motor behavior (Lagarde and Kelso, 2006; Harrison and Richardson, 2009; Sofianidis et al., 2012), which in turn might enhance beat perception (e.g., Phillips-Silver and Trainor, 2007), or by vibrotactile support of timbre and pitch perception (e.g., Russo et al., 2012). In general, it has been shown that tactile stimuli activate the auditory cortex, suggesting that physical contact might alter auditory perception (e.g., Schürmann et al., 2006). With regard to respiration and HRV, static hand holding increased inter-personal coupling in the context of pain research (e.g., Goldstein et al., 2017). In addition, indirect effects might benefit synchronization. For example, touch benefits emotional communication and increases social bonding (e.g., Hertenstein et al., 2006; for reviews see Gallace and Spence, 2010; Morrison, 2016).

**Figure 2:**
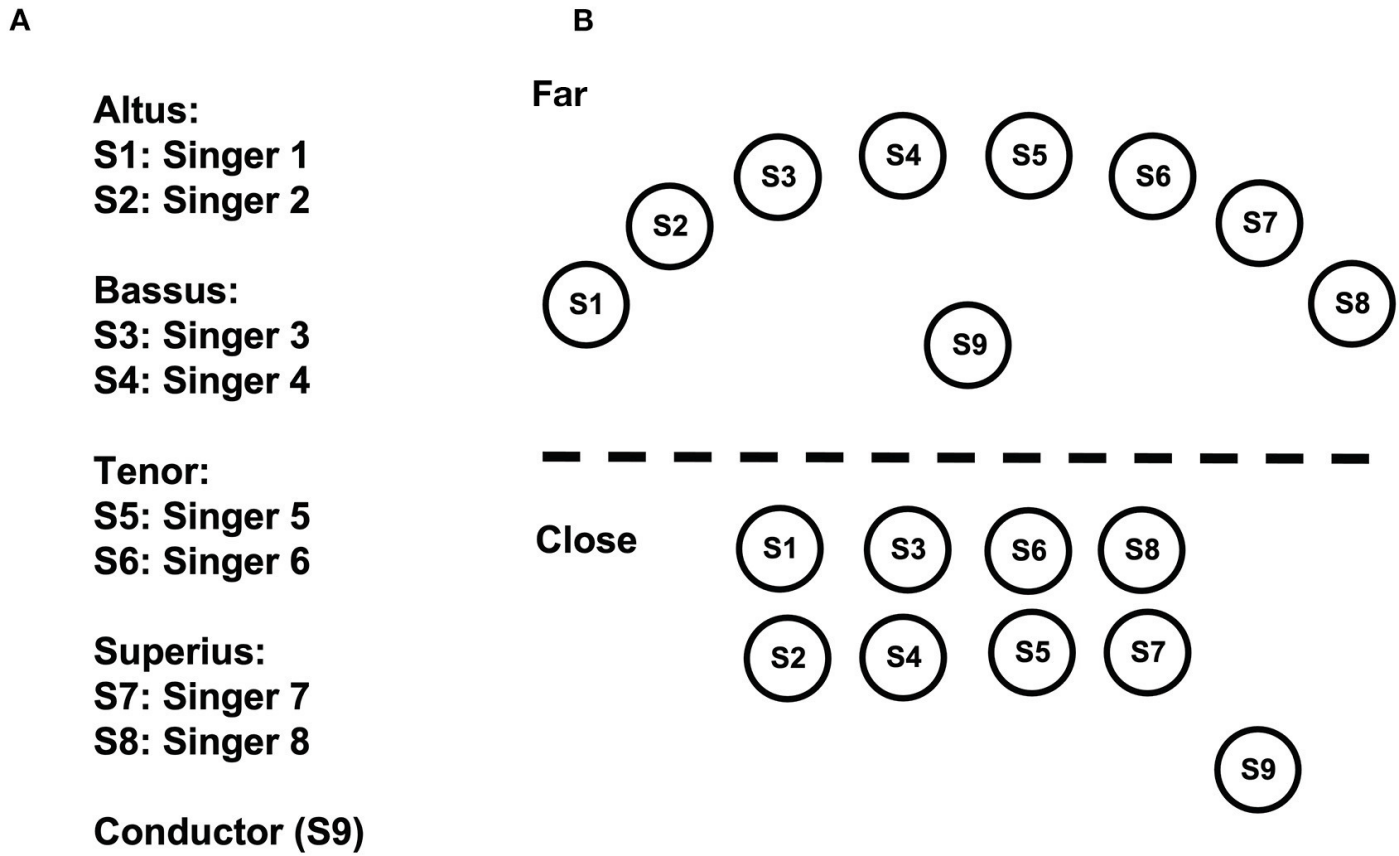
Configuration and assignment of the ensemble. The singers were placed in three standing conditions, with *ntf* relating to the depicted “far” positions, and *ntc* and *tc* to the close positions (see Methods for more information). **(A)** Assignment. **(B)** Configuration of the singing ensemble.

In our study, we compared the synchronization of respiration and HRV of professional singers performing polyphonic music from the Renaissance in three standing conditions: (i) Modern performance practice, distributed across the stage, each singer using their own music stand (no touch, standing far: *ntf* ); (ii) Standing close, all singers using one large music stand, with physical contact *via* putting the hand onto the neighboring singers (touch, standing close: *tc*); (iii) Standing close, using one music stand, but without physical contact as a control (no touch, standing close: *ntc*; see Figure 2). We applied a hyperscanning approach to measure respiration and HRV from all eight singers at the same time (Müller and Lindenberger, 2011). To avoid comparisons of single measures and their underlying potential confound, we repeated measures on three days in a balanced order. We predicted a stronger synchronization during singing in comparison to a resting condition, and an additional increase in synchronization during touch while singing. Further, we expected to see synchronization effects at different frequency bands of HRV.

## Materials and methods

### Participants

The ensemble consisted of six men and two women in addition to a male conductor with an age range of 29–45 years. The singers had 4–34 years of professional singing experience and were particularly experienced in Renaissance music, having sung such music for 3–30 years. The ensemble has a changing cast with, over the years, some members participating on a regular basis and others more infrequently. The singers in this study had been performing in the ensemble for a range of 1.5–12 years, and the conductor had taken the lead of the ensemble 12 years before our study commenced. The ensemble volunteered to participate in our study while they were on tour in Germany in May 2016 in exchange for accommodation expenses and a fee for their time. Data collection spanned three complete days.

### Apparatus

Physiology was acquired with a Brainamp ExG system. Three electrodes were placed on the chest of each of the eight singers in order to measure their heart rate: the first medially over the first rib between the two collarbones, the second left-lateralized just below the last rib, and the ground electrode next to the second but more medial. Respiration was measured by picking up the breathing extraction of the chest using a respiratory belt (BP-BM-10 by BrainProducts). This belt was placed on the upper chest but below the cables of the heart rate electrodes. No abdominal signal was recorded because particularly in singing both chest and abdomen breathing are coupled. The sampling rate at data acquisition was 1,000 Hz. Additionally to HRV and respiration, electroencephalography was acquired from two singers and the conductor, and motion capture was taken from the head of each singer and the conductor. The audio was recorded for all pieces. These additional measures are not part of the current analysis.

### Stimulus material

Pieces were selected from the repertoire of the ensemble: Josquin Desprez: the motets *Virgo prudentissima*, and *Tu solus qui facis mirabilia* (only second part: *D'ung aultre amer*); Guillaume Du Fay: *Kyrie, Gloria*, and *Agnus Dei* from the *Missa Ecce ancilla domini/Beata es Maria*. Pieces were arranged into three sections. In some cases, pieces or parts of pieces were repeated to reach sections of a minimum duration of 6 min. The sections contained (1) the two motets of Josquin in the order *Virgo prudentissima, D'ung aultre amer, Virgo prudentissima*; (2) *Agnus Dei* I, II, III, I, II; (3) *Kyrie* I, II, *Gloria*. In the following, we refer to the three sections as (1) *Motets*, (2) *Agnus Dei*, (3) *Kyrie/Gloria*. Modern notation was used to accommodate the choir's usual practice and to keep a high performance level.

For the most part, the chosen musical pieces represented the typical polyphonic style of the time, i.e., with imitative and free polyphonic passages and alternations of two-, three-, four-voices sections. However, the chosen musical pieces also included some homophonic passages (e.g., in *D'ung aultre amer*).

### Procedure

The day prior to data collection, the ensemble visited the laboratory concert hall to get instructed, practice the three standing positions, and give informed consent. Each of the three days started with setting up the participants with the physiology equipment, and on the second and third day, three of them with an EEG cap. The duration of preparation took 70–85 min. Then, as a practice run, all pieces were sung once in the same serial order (*Agnus Dei, Motets, Kyrie/Gloria*). This was followed by the experimental blocks, one for each of the pieces, and an additional resting control block on days one and three. However, due to technical problems we only collected two musical sections on day one (*Kyrie/Gloria* was excluded). In each of the experimental blocks three standing positions (conditions) were completed: (a) modern tradition: each singer with their own music stand, positioned in a semicircle, conductor centrally (*ntf* ); (b) historical tradition: all singers gathered close together in two rows of four, singing from one monitor (on which the sheet music was digitally presented instead of a music stand), having no physical contact, conductor placed next to them (*ntc*); (c) historical plus physical condition: Same as in (b) but with the physical contact of arms and hands (*tc*). Positions for ensemble members were marked on the floor for accuracy across repeated measures. However, as keeping positions and the physical contact points in (c) constant turned out to be too challenging and exhausting for the singers, they were allowed to adjust according to their needs on days two and three.

Due to technical problems, we diverged from the planned Latin Square of the serial order of pieces slightly but kept it for the standing positions. As a result, the serial positions of the pieces on day one were: rest, *Agnus Dei, Motets*; on day two were: *Motets, Kyrie/Gloria, Agnus Dei*; and on day three were: *Kyrie/Gloria, Agnus Dei*, rest, *Motets*. In total, we recorded 30 trials, based on eight musical blocks, two resting blocks, and each block in three standing conditions. Data collection took place from about 10 am to 4 pm. A longer lunch break was included and the ensemble indicated whenever they needed additional rest.

### Data preprocessing

We adjusted the length of each trial recording to be 360 s by clipping the end of the recording. Using BrainVision Analyzer software (Brain Products GmbH, Gilching, Germany), the QRS complexes in the ECG signals were identified and used for the determination of heartbeat locations. Once the timing of beats was determined, an instantaneous Heart Rate (HR) signal was created. Thereafter, HR and respiration signals were down-sampled to 10 Hz. Spencer's 15-Point Moving Average method was used to smooth a time series in order to highlight the underlying structure. Thereafter, mean and trends were removed from the HR and respiration data, and then the data were normalized to a unit variance. Note that heart rate variability (HRV) is determined by the time between heartbeats, known as RR intervals.

### Data analysis

The data analysis was strongly guided by an earlier study on synchronization of respiration and HRV of singers (Müller and Lindenberger, 2011). To investigate phase synchronization, we applied an analytic or complex-valued Morlet wavelet transform to compute the instantaneous phase in the frequency range from 0 to 1 Hz in 0.005-Hz steps. The complex mother Morlet wavelet, also called the Gabor wavelet, has a Gaussian shape around its central frequency *f* :


$$\begin{array}{l} \text{w}\left(\text{t, f}\right)={\left(\sigma^{2}\pi \right)}^{-1/4}exp\left(-{\text{t}}^{2}/2\sigma^{2}\right)exp\left(3/2\pi \text{jft}\right),\text{j}=\sqrt{-1} \end{array}$$

where σ is the standard deviation of the Gaussian envelope of the mother wavelet. The wavelet coefficients were calculated with a time step of 1, leading to a time resolution of 0.1 s.

In order to identify the phase relations between any two subjects/channels during the task, the instantaneous phase difference Δϕ_*mn*_(*t, f* ) was computed from the wavelet coefficients for all possible subject/channel pairs. Three different synchronization measures were obtained from these phase differences for frequency of interest (FOI) *f*_*i*_. Initial power spectral density (PSD) analyses showed no clear peaks and therefore provided no guideline for selecting relevant FOIs. We therefore decided to use the same ten frequencies as in an earlier study (Müller et al., 2018a), which were chosen with regard to the fixed relation between frequencies (1:2, 1:3, 2:3, etc.): 0.025, 0.05, 0.075, 0.10, 0.125, 0.15, 0.20, 0.25, 0.30, and 0.40 Hz. These ten frequency components practically cover the whole frequency spectrum of breathing and HRV during singing.

We obtained the Phase Synchronization Index (PSI), which is defined as the mean vector length of the angular dispersions of the phase difference in a complex space. It was calculated by PS$I_{\Phi }\left(f_{i}\right)=|\langle e^{j\cdot \Delta \Phi^{k}\left(f_{i}\right)}\rangle |,j=\sqrt{-1}$, where $\Delta \Phi^{k}=\;\mathrm{mod}\;\left(\Phi_{m}^{k}\left(f_{i},t\right)-\Phi_{n}^{k}\left(f_{i},t\right),2\cdot \pi \right)$, which is the phase difference with instantaneous phases of these two signals across k data points during the task condition; $\Phi_{m}^{k}\left(f_{i}\right)=\arg\left\{y_{m}^{k}\left(f_{i}\right)\right\}$ and $\Phi_{n}^{k}\left(f_{i}\right)=\arg\left\{y_{n}^{k}\left(f_{i}\right)\right\}$. The PSI is most widely used in research applying electroencephalography (Müller et al., 2013; Hemakom et al., 2017; Borovik et al., 2020) but also with respiration and HRV (Müller and Lindenberger, 2011).

With the estimates of the phase difference between pairs of signals (participants), it is then possible to ascertain how long the phase difference remains stable in defined phase angle boundaries by counting the number of points that are phase-locked at a defined time window. Analogous to Müller and Lindenberger (2011; see also Kitzbichler et al., 2009), we divided the range between -π/4 and +π/4 into two ranges, one marked the negative deviations in the range between -π/4 and 0 (coded with “−1,” see blue color in **Figure 5**), the other the positive deviations in the range between 0 and +π/4 (coded with +1, see red color in Figure 5). Phase differences beyond these ranges represent non-synchronization (coded with “0,” see green color in Figure 5). By counting the relative number of phase-locked points within the range −π/4 and +π/4, we obtained the Absolute Coupling Index (ACI). In addition, we derived the Integrative Coupling Index (ICI), which combines information of the ACI and the relative number of phase-locked point within the positive range (Positive Coupling Index, PCI) and is an asymmetric coupling measure: ICI = ((PCI + ACI)/(2^*^ACI)${)}^{*}\sqrt{\text{PCI}}$. The ICI equals 1, when all phase-locked points are in the positive range, and zero, when they are in the negative range.

We report results on the three coupling measures PSI, ACI, and ICI. To determine the effects of singing and touch on the coupling of respiration and HRV measures across participants, we made use of two-way repeated measures ANOVAs with the factors Frequency and Condition, comparing either singing to rest in *ntf* , or touch to no touch in *tc* and *ntc*. We concentrated on these two comparisons because for an effect of singing, we did not want to include the rather unusual close standing conditions (*tc* and *ntc*), while for an effect of touch, the regular standing condition *ntf* is not a suitable control. We grouped the ten frequencies in three ranges that relate to specific physiological processes. Very low frequencies (VLF: 0.025, 0.05 Hz) and low frequencies (LF: 0.075, 0.10, 0.125, 0.15 Hz) support the sympathetic nervous system, and high frequencies (HF: 0.20, 0.25, 0.30, 0.40 Hz) the parasympathetic. We applied the Greenhouse-Geisser epsilon for non-sphericity, wherever necessary. We used IBM SPSS v25 (SPSS, Chicago, IL, USA) for statistical analysis. In addition to rigor testing, we explored the relationship between coupling and musical structure in a descriptive way, looking into dynamic changes across time.

## Results

### Effect of singing on synchronization

**Figure 3:**
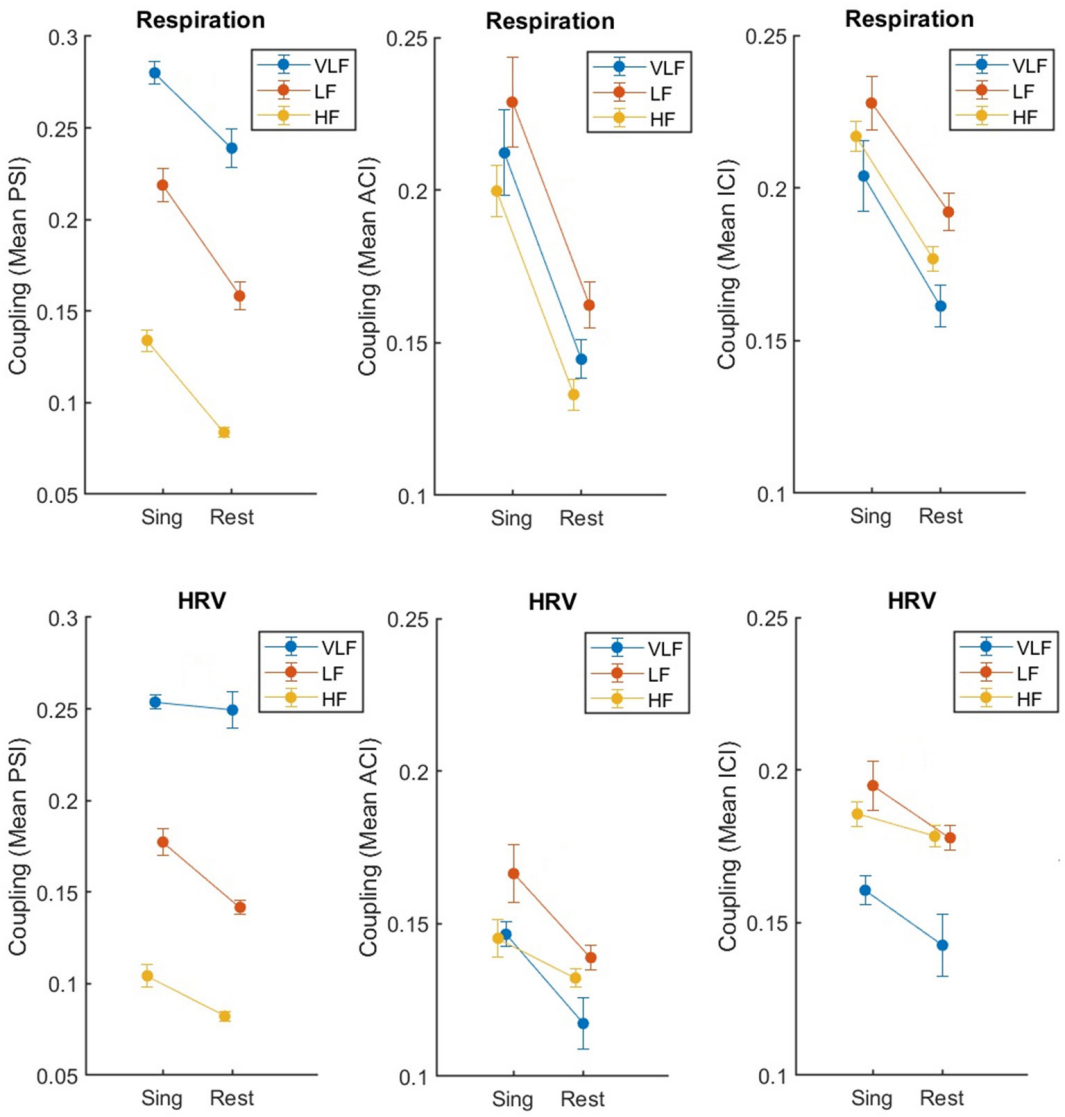
Synchronization of respiration (upper part) and HRV (lower part) between eight singers recorded during singing or resting (both in standing condition *ntf* ), measured by mean PSI, ACI, and ICI (from left to right). The error bars depict the standard error of the means.

The two-factor ANOVA with the factors Condition (singing vs. rest) and Frequency (VLF, LF, HF) included data from the regular standing position only (*ntf* ) to avoid any confounding effects of standing close and having physical contact. Table 1 lists the statistical results for all three coupling indices: PSI, ACI, and ICI. The results are clear. The factor Condition was significant as well as the factor Frequency, with the interaction being not significant (with one exception: HRV, PSI; pairwise *post-hoc* comparisons revealed that the interaction was due to a missing effect of singing for VLF, *p* > 0.10). Figure 3 shows that, indeed, the coupling of respiration and HRV was higher during singing than at rest, and this effect was similar across different frequency ranges.

**Table 1:** Results of the two-factor ANOVA on the effect of singing.

		**Condition (singing, rest)**			**Frequency**			**Cond**. * **Frequ**.		
		**(*****df*** = **1, 7)**			**(*****df*** = **2, 14)**			**(*****df*** = **2, 14)**		
		***F***	***p***	**η^2^**	***F***	***p***	** $\eta_{\text{p}}^{2}$ **	***F***	***p***	** $\eta_{\text{p}}^{2}$ **
Respiration	PSI	37.44	<0.001	0.842	432.31	<0.001	0.984	1.89	0.201	0.213
	ACI	28.30	0.001	0.802	13.41	0.002	0.657	0.011	0.958	0.002
	ICI	21.39	0.002	0.753	10.82	0.007	0.607	0.35	0.705	0.048
HRV	PSI	9.22	0.019	0.568	338.27	<0.001	0.980	7.74	0.017	0.525
	ACI	30.57	0.001	0.814	9.23	0.006	0.569	1.17	0.326	0.143
	ICI	27.25	0.001	0.796	38.64	<0.001	0.847	0.442	0.554	0.059

### Effect of touch on synchronization

**Figure 4:**
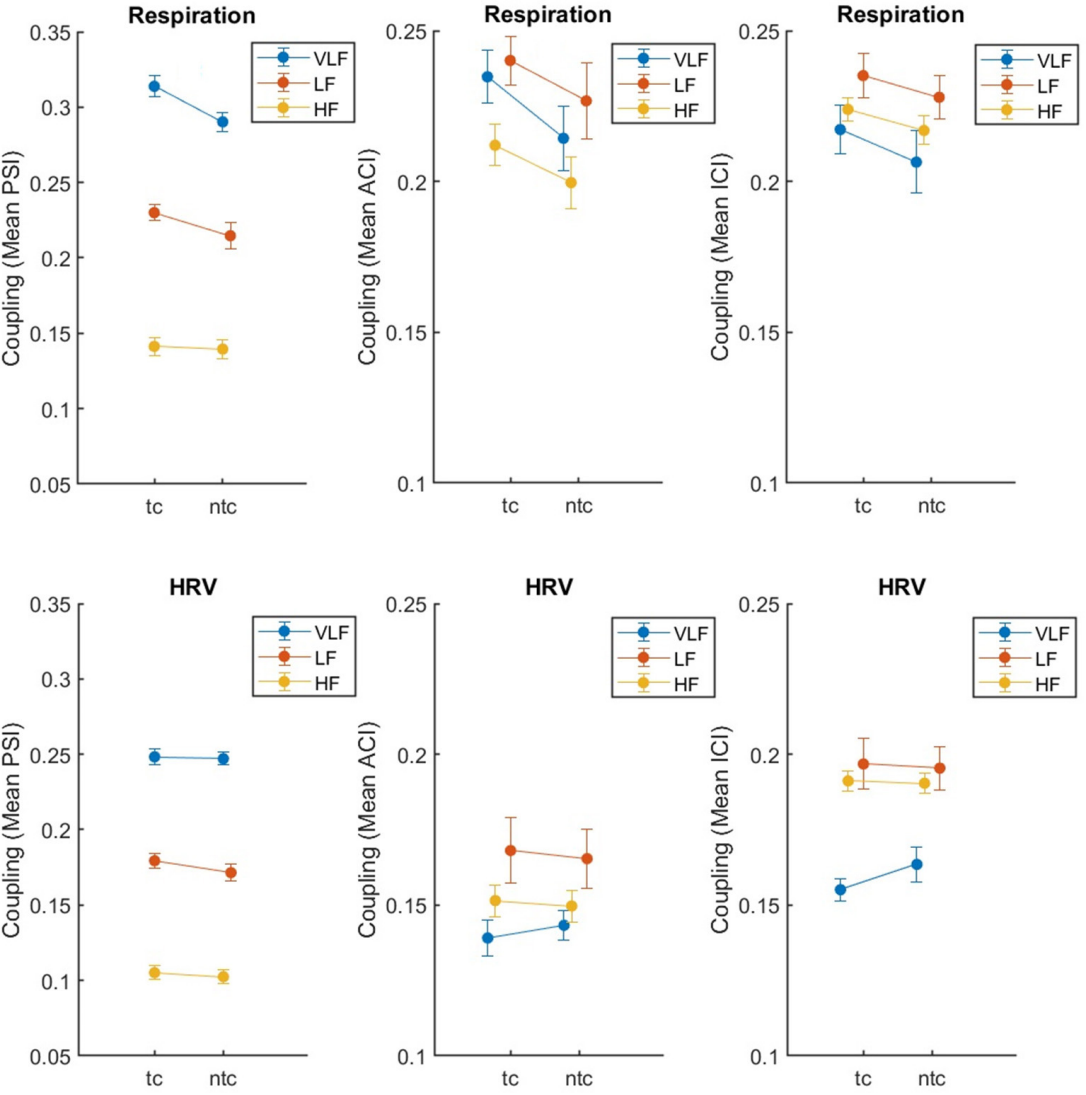
Synchronization of respiration (upper part) and HRV (lower part) between eight singers recorded during singing with (*tc*) and without (*ntc*) physical contact, measured by mean PSI, ACI, and ICI (left to right). The error bars depict the standard error of the means.

We tested for the effect of touch, comparing synchronization of respiration and HRV in the two close standing conditions, with and without touch (*tc, ntc*). Again, the results are clear (see Table 2). For respiration, there was an effect of touch (significant for PSI and ACI, and a tendency with *p* < 0.10 for ICI), while for HRV there was no such an effect (all *p*'s > 0.11). The main effect of Frequency was significant for all three measures and both physiological recordings. None of the two-way interactions reached significance (but there was a tendency with *p* < 0.10 for PSI in respiration; the *post-hoc* pairwise comparison indicated no difference between touch conditions for HF, *t* < 1). Overall, Figure 4 shows that the effects were as expected: Singing with physical contact resulted in a higher coupling of respiration than singing without. For HRV, some tendencies in the same direction were revealed but were far from significant (i.e., for PSI *p* = 0.113).

**Table 2:** Results of the two-factor ANOVA on the effect of touch.

		**Condition (** ***tc, ntc*** **)**			**Frequency**			**Cond**. * **Frequ**.		
		**(df** = **1, 7)**			**(df** = **2, 14)**			**(df** = **2, 14)**		
		***F***	***p***	**η^2^**	***F***	***p***	** $\eta_{\text{p}}^{2}$ **	***F***	***p***	** $\eta_{\text{p}}^{2}$ **
Respiration	PSI	8.17	0.024	0.539	297.91	<0.001	0.977	4.95	0.051	0.414
	ACI	9.54	0.018	0.577	20.29	<0.001	0.743	1.15	0.329	0.141
	ICI	3.96	0.087	0.361	6.08	0.020	0.465	0.26	0.718	0.036
HRV	PSI	3.28	0.113	0.319	576.85	<0.001	0.988	1.18	0.333	0.144
	ACI	0.09	0.769	0.013	7.85	0.016	0.529	1.69	0.228	0.194
	ICI	0.135	0.724	0.019	26.16	<0.001	0.789	1.14	0.329	0.140

### Changes in synchronization across time

**Figure 5:**
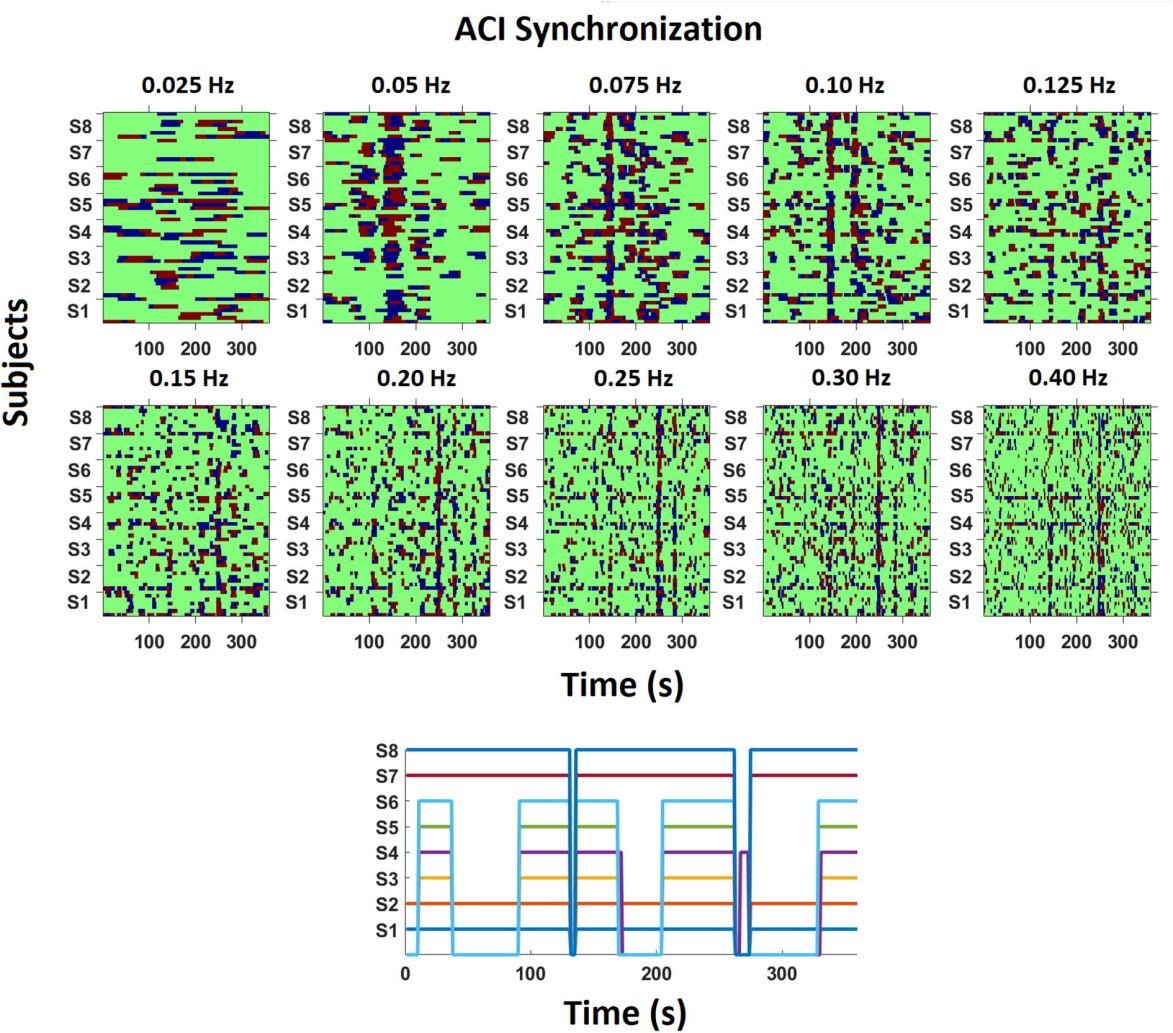
Example of phase synchronization patterns of respiration at different frequencies across 360 s. The ACI between each singer (S1–S8) and every other singer was calculated pairwise for ten frequency bins. Red depicts that one singer's oscillation is leading, blue that the other singer is leading, and green depicts no synchronization within the defined range of the phase difference (see Method for more information). In this recording, the ensemble was singing the *Kyrie/Gloria* in *tc* on the 3rd day. The subplot on the bottom depicts which singer was singing across time, with singers S1, S2, S7, and S8 singing throughout the musical piece, and S3 to S6 having longer passages without singing.

Figure 5 depicts examples of the synchronization of respiration across time for all frequencies from one recording (Kyrie/Gloria in *tc* on day three). On a descriptive level, it is obvious that the synchronization of an ensemble singing a polyphonic piece is less stable across time than what has previously been reported with an amateur choir singing canons (see Müller and Lindenberger, 2011, Figure 3). In our data and this specific example, one can visually identify several time intervals showing synchronization between singers across different frequencies. For instance, there is high pairwise synchronization across most singers in the time interval 130–150 s for periodicities around 0.05, 0.075, 0.10, 0.125, and 0.40 Hz, with some synchronization (but not across all singers) for 0.15, 0.20, 0.25 and 0.30 Hz. Further, a second time window of 240–260 s shows strong synchronization in the higher frequencies from 0.125 to 0.40 and some synchronization also for lower frequencies. That is, synchronization occurs for a diverse range of frequencies and is not limited to a specific frequency. Synchronization also occurs for different frequency ranges at different time points. Note that the two time intervals mentioned above, are related to musical sections such as the beginning of Kyrie II (at around 137 s) and the end of Kyrie II (240–260 s). The beginning is special as after a short break all singers start simultaneously for the first time in that piece and then sing relatively homophonous for a few measures. In addition, the entry of the cantus firmus in the Tenor (“Beata es Maria”) marks the first musical climax in Du Fay's *Missa Ecce ancilla domini/Beata es Maria*, and was also performed with particular emphasis by the singers of the tenor. The end has a purely chordal, i.e., homophonic structure with chords that change only very slowly. That is, the compositional structure is revealed in the synchronization pattern.

We also see differences between singers regarding their synchronization. For instance, in the time window of 180–200 s and at the frequency of 0.075 Hz the subgroup of singers S3–S8 was connected with each other, but connections from S3–S8 to S1 and S2 were missing, whereas S1 and S2 were coupled. However, at this time S1 and S2 as well as S7 and S8 were singing in a duo, with S1 and S2 singing one voice (Altus) and S7 and S8 the other (Superius). S3–S6 listened (see also Figure 5 lower subplot, depicting singing activity for each singer across time). It is interesting that S3–S6 (Bassus, Tenor) were connected to S7 and S8 (Superius) and not to S1 and S2 (Altus). There seems to be no musical explanation for preferably connecting with the Superius. Importantly, synchronization is not systematically organized by the singing action but also occurs between singers and listening ensemble members. As a further example, at the frequency of 0.25 Hz (and other frequencies), horizontal lines reflect a sustained synchronization between S1 and S2, S4 and S5 as well as S7 and S8. While S1 and S2 as well as S7 and S8 were actually singing the same voice, S4 and S5 were not, but rather stood in neighboring positions and were connected by touch (see Table 3). Hence, for this pair (S4, S5) not singing together but touching each other seemed to increase synchronization. Note, too, that the connectivity was intermittent for S1 and S2, as well as S7 and S8, even though these singers were singing most of the time (i.e., compare the vertical, intermittent line patterns in the upper part of Figure 5 with the depicted singing activity in the lower subplot of Figure 5).

**Table 3:** Overview of the singers' neighboring positions and realized touch conditions in the trial depicted in Figure 5.

**Singer**	**Neighbor**	**Physical contact**
S1	S2, S3, S4	• Touched hands with S2, • Touched shoulder of S3
S2	S1, S3, S4	Touched hands with S1
S3	S1, S2, S4, S5, S6	• Touched waist of S1, • Touched shoulder of S4
S4	S1, S2, S3, S5, S6	Touched waist of S5
S5	S3, S4, S6, S7, S8	• Touched waist of S4, • Touched shoulder of S7
S6	S3, S4, S5, S7, S8	• Touched waist of S3, • Touched waist of S1
S7	S5, S6, S8	Touched shoulder of S5
S8	S5, S6, S7	Touched waist of S6

To further explore how synchronization in respiration was related to the fact that all singers were singing or not, we decided *post-hoc* on a comparison between synchronization measures during time intervals when all singers were singing in comparison to when only part of the ensemble was singing. Given the observations above, synchronization should be stronger for passages with all singers in comparison to only part of it. Regarding the effect of touch, both outcomes are possible: A stronger effect of touch when part of the ensemble was singing. Eventually, touch is particularly effective when there is no other means for coordination like singing; or, a stronger effect of touch when all are singing, as the joint action of singing might be the base for physical contact to be effective.

**Figure 6:**
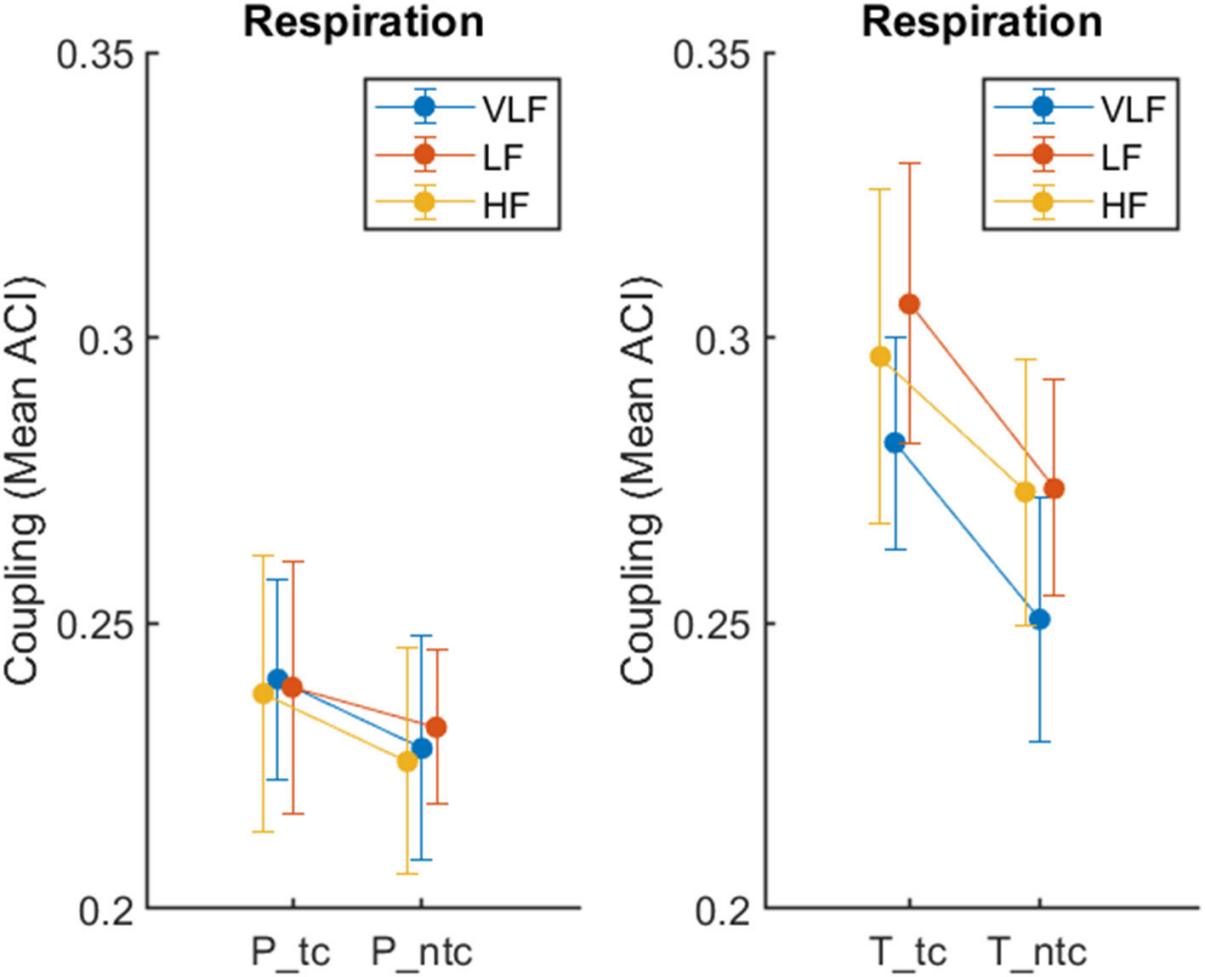
Synchronization of respiration during passages when only part of the ensemble was singing (P: partial, left) in comparison to all singers (T: total, right), recorded during singing with (*tc*) and without (*ntc*) physical contact, measured by ACI. The error bars depict the standard error of the means.

For this analysis, we report on ACI as a synchronization measure for respiration only and focus on recordings from the conditions *ntc* and *tc*. We annotated which singer was singing within the 360 s recordings, based on seconds as time unit. We marked sequences when all singers were singing (condition: total). To keep the lengths of the passages about the same, we compared these sequences with times, when the number of active singers was below eight but not zero (condition: partial). We dropped time units, when only a single unit differed from the other surrounding ones (e.g., 1 s, in which only seven singers instead of eight were singing). The mean number of time units across all recordings in the condition total was 168 s (*SD* = 10) and for partial 142 s (*SD* = 14). We then calculated the mean ACI in the same way than before, but separately for total and partial singing. We fit the data into a three-way ANOVA with the within-subject factors Touch (*ntc, tc*) and Ensemble (*total, partial*), and Frequency (VLF, LF, and HF). The main effect of touch was significant, *F*_(1,7)_ = 22.71, *p* = 0.002, $\eta_{\text{p}}^{2}$ = 0.764, with touch resulting in higher synchronization (*M* = 0.27, *SE* = 0.02) than no touch (*M* = 0.25, *SE* = 0.02). The main effect of ensemble was significant, *F*_(1,7)_ = 191.13, *p* < 0.001, $\eta_{\text{p}}^{2}$ = 0.965, with synchronization being higher for total (*M* = 0.28, *SE* = 0.02) in comparison to partial (*M* = 23, *SE* = 0.02). The main effect of frequency failed to be significant (*p* = 0.059). The two-way interaction ensemble-by-touch was significant, *F*_(2,14)_ = 19.45, *p* = 0.003, $\eta_{\text{p}}^{2}$ = 0.735. Figure 6 shows that the effect of touch was stronger for total than partial. In addition, the two-way interaction ensemble-by-frequency was significant, *F*_(2,14)_ = 35.37, *p* < 0.001, $\eta_{\text{p}}^{2}$ = 0.835, which was based on a slightly smaller effect of ensemble for VLF than for LF and HF.

To test whether the effect of touch was still present when only part of the ensemble was singing, we limited the analysis on the two factors Touch and Frequency and the data from the partial condition. The main effect of touch only showed a non-significant tendency, *F*_(1,7)_ = 4.75, *p* = 0.066, $\eta_{\text{p}}^{2}$ = 0.404. The effect of frequency was far from significant as well as the interaction, both F's <1.

## Discussion

Our results confirm previous findings on synchronization of respiration and HRV during ensemble singing and extend those findings by revealing an increase in synchronization under physical contact. Singing increased the coupling between singers in comparison to rest in both respiration and HRV. This effect was significant across the different frequency ranges. With these results, we replicate the findings by Müller and Lindenberger (2011). Importantly, we show that singing synchronizes respiration and HRV, even in the current setup where a professional ensemble is singing polyphonic music with distributed breathing. We also extended previous findings by showing that singing with touch led to higher synchronization than singing without touch. This effect became significant in respiration but not for HRV. Moreover, the effect of touch was particularly pronounced during parts when all singers in the ensemble were singing at the same time in comparison to the partial ensemble.

As this study breaks new ground regarding joint singing with touch, parallels can only be drawn to studies of the effects of physical contact on other behaviors. The synchrony effects of touch during singing might stem from a higher activation of the auditory cortex through the vibrotactile support (e.g., Schürmann et al., 2006), which in turn enhances support of timbre and pitch perception (e.g., Russo et al., 2012). This might explain, why the effect of touch was particularly effective when the full in comparison to the partial ensemble was engaged in the joint action of singing. In addition, physical contact such as hand-holding has been shown to increase respiration in inter-personal coupling (e.g., Goldstein et al., 2017), indicating that the current measures reflect these processes. Of course, these theses would need further support from brain imaging techniques. In any case, touch has various (indirect) effects on people which might benefit synchronization through (emotional) communication and social bonding (e.g., Hertenstein et al., 2006, for reviews see Gallace and Spence, 2010; Morrison, 2016), but might also hinder synchronization in situations where people do not want physical contact or their natural movement behavior is too restricted by maintaining contact.

Some observations from the descriptive time analyses need to be discussed. Firstly, we see that synchronization in our study is less stable across time than what has previously been reported by Müller and Lindenberger (2011). One reason might lie in the music selection, another in the experience level of the singers. While we selected highly intricate polyphonic music, in other studies the music was chosen because of its simple structure (i.e., Vickhoff et al., 2013), clear tempo, and distinct phrase endings. With these simple stimuli, tempo and breathing rhythm are related strongly to the synchronization of specific frequencies in respiration and HRV. On a side note, we looked into a potential relation between musical tempo and the respiration signal in our own data, but did not find supporting evidence. Also, singing in unison resulted in higher coupling than singing a canon in parts (Müller and Lindenberger, 2011), indicating that the lower complexity of the music has a positive impact on synchronization. In addition, we invited an ensemble with professional singers that applied distributed breathing, whereas the study from Müller and Lindenberger (2011) involved an amateur choir. Both the complex musical structure and distributed breathing onsets might have lowered synchronization between singers in our study.

Looking at the changes in synchronization over time, synchronization was seen to occur for a range of frequencies at various moments. In some of these moments, all singers were synchronized, in some only subgroups were synchronized. While this can in some cases be explained by the musical structure (homophonic vs. polyphonic parts, only some voices singing vs. all voices singing), most of the time, synchronization was not systematically organized by the singing action (e.g., singing the same voice), but also occurred between active and “passive” singers, that is those resting at certain points. While research has shown that coupling between singers is higher than between members of the audience (Hemakom et al., 2016), we see that listening as a singer is a very engaging activity as singers who rest need to follow the other singers in order to get the cue for their next entry. Hence, subgrouping is not simply due to the fact of singing being contrasted with non-singing activity. Here, the musical experience might also come into play. It might very well be that a professional singing ensemble is able to create such a superordinate system, indicated by the strong coupling between active singers and the other singers of the ensemble (cf. Müller et al., 2018a).

However, we indeed found an effect of total vs. partial ensemble singing in our *post-hoc* analyses. Synchronization of respiration was higher, when all singers sang, in comparison to only part of the ensemble. Note that most of the time the music was polyphonic when the full ensemble sang, with a complex structure and distributed breathing. Then, this analysis does not compare homophonic with polyphonic singing—an analysis, which was not possible, because the homophonic parts were rather rare and data then too sparse.

Synchronization was present across different frequency ranges at the same time, spanning very low to high frequency bands. From a physiological perspective, VLF and LF support the sympathetic nervous system and HF the parasympathetic. There is also evidence that LF can be modulated by both sympathetic and parasympathetic activities (Ernst, 2017). Then, synchronization was present in the sympathetic and parasympathetic nervous systems and was not limited to either of the two. Whereas, the sympathetic system controls the dilation of the bronchi and acceleration of the heart rate, the parasympathetic system constricts the bronchi and slows the heart rate (Eckberg, 2000). As reported (Bonsignore et al., 1995; Yasuma and Hayano, 2004), heart rate increases at inspiration and decreases at expiration, reflecting respiratory-circulatory interactions. Heart rate variability in synchrony with respiration is a biological phenomenon known as respiratory sinus arrhythmia (RSA), playing a role in the HRV coupling occurring in singing interactions (Müller and Lindenberger, 2011; Vickhoff et al., 2013).

As the current project was interdisciplinary in nature, historical sources that pointed to the practice of singing with body contact were of interest. However, since pictorial representations cannot be taken at face value and corroborating sources are missing, one cannot say for sure whether historical ensembles really touched during their rehearsals and performances. A reason for touch in medieval and early modern times might have been the need to ensure high-precision coordination among singers in the absence of cues in the music notation and a conductor. Scholarship on the cultural history of touch (Classen, 2012), cultural anthropological studies on group singing worldwide (Hayward, 2014), but also results from social psychological research on the effects of interpersonal touch on action coordination and feelings of social connectedness (Gallace and Spence, 2010; Cekaite and Mondada, 2020) make it seem plausible to assume that physical contact and vibrotactile perceptual input can serve as a source of coordination and entrainment during joint singing. The current findings support these claims as touch increased connectivity during singing, at least for respiratory activity.

However, as touch only added to the already strong increase in connectivity that was seen from rest to singing, the function of touch in the current study needs to be discussed. Different from Renaissance practice, the current ensemble sang from modern scores and had a conductor who took over the coordination. Hence, in our study, touch did not need to serve an intentional function besides following the instructions of the experimenter, which may explain the smaller synchrony effect for touch vs. no touch. Touch is a multidimensional, socially coded behavior and communicates a variety of contents, like love, intimacy, bonding, solidarity, friendship, comfort, sexual intention, aggression, dominance, status, or power (Hertenstein et al., 2006), via a variety of features, such as duration, intensity, location, etc. (Major, 1981). There are strong inter-individual differences in the ways people feel and interpret touch, particularly outside a romantic relationship (Major, 1981). In a context like ours, where the singers were asked to maintain physical contact, singers might have a wide range of reactions, with some of the singers feeling more pleasure and others irritation (as some singers of our ensemble actually mentioned after data collection). This divergence might have contributed to inter-individual differences in physiological coupling via touch. Forcing singers to maintain physical contact also created some difficulties, e.g., the radius of free movements was restricted, and touching while concentrating on singing might have created some difficulties, and hence dual-task costs (onto the singing performance). One might speculate whether touch might have been more disturbing during passages when only part of the ensemble was singing than when the ensemble was unified in the joint action of singing. However, despite potential difficulties, the coupling increased as a result of physical contact, showing a general benefit of touch for respiration synchronization.

Our study showed increased physiological synchronization between singers. However, it is difficult to disentangle what exactly drove synchronization. Joint action requires several processes and representations to overlap between co-actors (Vesper et al., 2017), such as the mental representation of action goals and monitoring task progress, the sharing of sensorimotor information including ongoing multisensory perceptual and emotional processes, sensorimotor predictions of the own and others' actions, and general mechanisms supporting coordination. However, we tentatively argue that some simple underlying reasons for synchronization can be ruled out. We see mixed patterns of synchronization between singers that could not be explained by either singing the same voice, having physical contact or spatial distance between singers. Importantly, therefore, our findings suggest a higher level of organization of all singers, forming a superordinate system (Noble, 2012) or superorganism, here in the form of a music ensemble (Müller et al., 2018a, 2019). With this, we propose that our findings follow the theoretical model of the human supersubject (Müller et al., 2021) reflected in professional ensemble members who are familiar with each other and the sung music, and that share the same goals.

## Conclusion

While it had previously been shown that joint singing increases the synchronization of respiration and HRV, the current study extends these findings by revealing an additional increase in synchronization of respiration when singing with body contact. By taking an interdisciplinary approach, the current study stands out in the field of synchrony during singing. Investigation of singing with physical contact was inspired by historical accounts and the professional ensemble recruited sang intricate polyphonic music. We showed that synchrony increased even under these highly specific circumstances. Interestingly, synchrony was not shown to be systematically related to the singing activity such as singing the same voice, or either standing position or touch. The ensemble seemed to organize itself on a higher level, possibly creating a superordinate system where singers share the same goals.

## Data availability statement

The datasets presented in this study can be found in online repositories. The names of the repository/repositories and accession number(s) can be found at: https://osf.io/tejvy/.

## Ethics statement

All procedures were conducted in accordance with the 1964 Helsinki Declaration and its later amendments, and approved by the Ethics Council of the Max Planck Society (number 2702_12). All participants provided their written informed consent to participate in this study.

## Author contributions

MW-F conceived the research. EL, DO, and JM designed the research. EL, DO, MW-F, and JM collected the data. EL, DO, CT, and VM performed the analyses. DO, CT, and VM contributed to the Methods and Results Sections. EL and JM wrote the manuscript. All authors revised the manuscript. All authors contributed to the article and approved the submitted version.

## Funding

This research was supported by the Max Planck Society.

## Conflict of interest

The authors declare that the research was conducted in the absence of any commercial or financial relationships that could be construed as a potential conflict of interest.

## Publisher's note

All claims expressed in this article are solely those of the authors and do not necessarily represent those of their affiliated organizations, or those of the publisher, the editors and the reviewers. Any product that may be evaluated in this article, or claim that may be made by its manufacturer, is not guaranteed or endorsed by the publisher.

## Footnotes

^1^With voice, we are referring to the individual voices of a polyphonic composition, e.g., soprano, alto, tenor, and bass.
