# Supplementary material for: In touch: Cardiac and respiratory patterns synchronize during ensemble singing with physical contact
Source: Front Hum Neurosci. 2022 Aug 5;16:928563. doi: 10.3389/fnhum.2022.928563 (PMC9390082; doi:10.3389/fnhum.2022.928563)
Supplement: Supplementary file 1 [file Data_Sheet_1.pdf]

Supplementary Material

[Supplement to: Lange, E.B., Omigie, D., Trenado, C., Müller, V., Wald-Fuhrmann, M., & Merrill, J., *In touch: Cardiac and respiratory patterns synchronize during ensemble singing with physical contact,* Frontiers in Human Neuroscience, 2022]

Inter-subject correlation of breathing onsets

Müller and Lindenberger (2011) showed synchronization of respiration and HRV between singers singing a canon in unison or with distributed voices. We assume that part of the synchronization might be due to the fact that singers inhaled air at the same time due to musical restrictions and the fact that the choir were laypersons. In favor of this argument, Vickhoff et al. (2013) showed that singing a mantra with instructed breathing at 0.1 Hz resulted in a peak of 0.1 Hz in the respiration and HRV signal. For our study, it was important to us to invite a professional choir, singing highly complex polyphonic music with a distributed breathing technique. Such distributed breathing is performance practice with professionals to make the sounds blend into a continuous stream. To test whether the members of our choir indeed sang with distributed breathing, we analyzed cross-correlations between singers. We manually coded breathing of each singer individually, based on the audio recordings, with a resolution of 1 s. We analyzed the audibel breathing onset for inter-subject synchronization and did not find any cross-correlations. Hence, the singers did not breathe at the same time. Figure S1–S3 depicts the cross-correlation (CC) function of breath onsets between different pairs of singers, separately for the three days of data collection, some pairs singing the same voice ((1,2), (3,4), (5,6), (7,8)), and others not ((2,3), (4,5), (6,7)). Here, CC quantifies the number of coincidences in a pair of time series (breathing onsets) for different lags. For instance, on day 1 between singers 1 and 2 at lag zero there were 25 coincidences; between 3 and 4 there were 35 coincidences, between 7 and 8 there were about 32 coincidences. It is noticeable that pairs (1,2), (3,4) and (7,8) have a clear peak at lag zero and have more similar breath onsets but not equal, which is apparent by considering the asymmetry of the CC graphs. The fact that there are coincidences at different lags highlights differences in breath onsets between singers in a pair. Note that pairs (2,3), (4,5), (5,6), (6,7) show less coincidence between breath onsets. Importantly, the same pattern was highly similar during days 2 and 3, with some exceptions particularly for the pairs singing with the same voice: the number of coincidences at lag zero decreased (i.e., see difference between day 1 and 2 for (1,2), (3,4), (7,8)), and the peak at lag zero was less unique (i.e., compare day 2 and 3 for (3, 4), (5, 6)), and the distributions became less dense. Whereas the last observations seem to show lower numbers of audible breathings overall, the first two observations demonstrate increased distributed breathing for consecutive days of singing practice.

Figure S1.

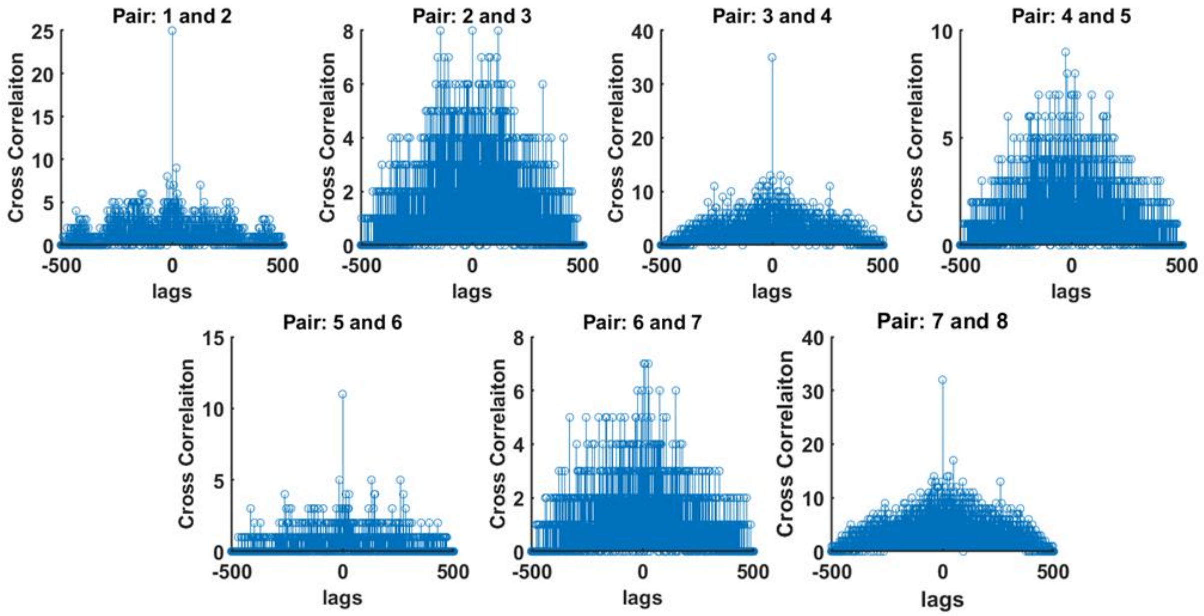

Subplots depict pairwise cross-correlations between singers on day 1 of three consecutive days of recordings, here coded as pairs, e.g., Pair 1 and 2 refer to singers S1 and S2.

Figure S2.

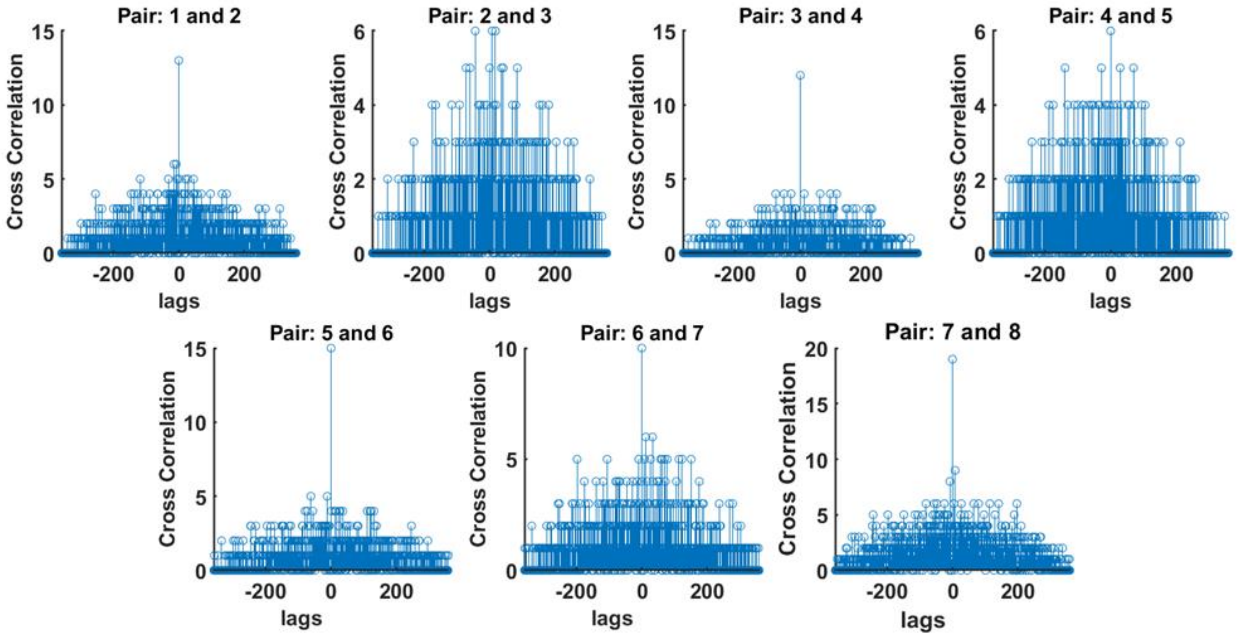

Subplots depict pairwise cross-correlations between singers on day 2 of three consecutive days of recordings, here coded as pairs, e.g., Pair 1 and 2 refer to singers S1 and S2.

Figure S3.

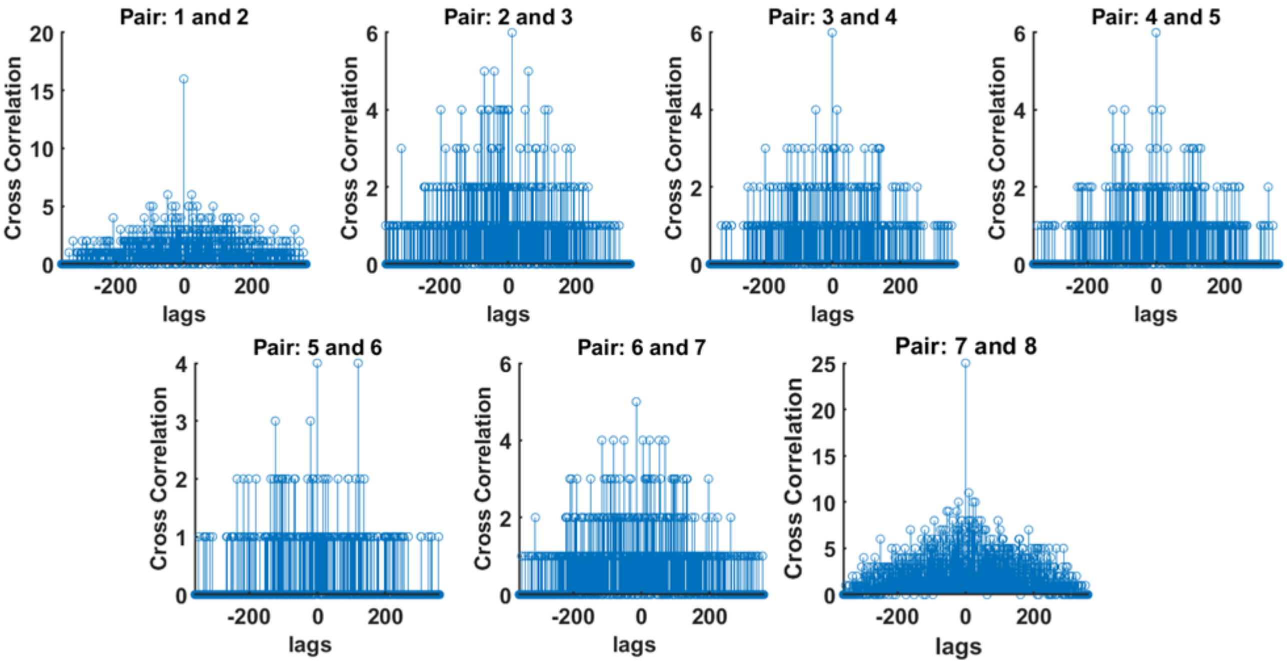

Subplots depict pairwise cross-correlations between singers on day 3 of three consecutive days of recordings, here coded as pairs, e.g., Pair 1 and 2 refer to singers S1 and S2.
